# Dietary protein to starch metabolizable energy ratios alter growth performance and gastrointestinal microbiota of calves

**DOI:** 10.3389/fmicb.2023.1065721

**Published:** 2023-03-01

**Authors:** Xue Chen, Zhiyi Han, Jianan Dong, Jun Xiao, Wei Zhao, Jiye Rong, Natnael D. Aschalew, Xuefeng Zhang, Guixin Qin, Yuguo Zhen, Zhe Sun, Tao Wang

**Affiliations:** ^1^Key Laboratory of Animal Nutrition and Feed Science of Jilin Province, Key Laboratory of Animal Production Product Quality and Security Ministry of Education, College of Animal Science and Technology, JLAU-Borui Dairy Science and Technology R&D Center, Jilin Agricultural University, Changchun, China; ^2^Postdoctoral Scientific Research Workstation, Feed Engineering Technology Research Center of Jilin Province, Changchun Borui Science and Technology Co., Ltd., Changchun, China; ^3^College of Life Science, Jilin Agricultural University, Changchun, China

**Keywords:** blood index, calves, diet structure, growth and development, microbiome analysis

## Abstract

The diet structure is very important for the growth and development of calves. This study aimed to investigate the effects of dietary protein-to-starch metabolizable energy ratios (DPSRs) on growth performance, blood index, and gastrointestinal microbiota of calves. Forty-eight Holstein bull calves were fed six dietary DPSRs including A20-35 (20% CP and 35% starch), B20-30, C20-25, D22-35, E22-30, and F22-25 at d 4 to d 60, and then changed to another six dietary DPSRs at d 61 to d 180 (A18-30, B18-27, C18-24, D20-30, E20-27, and F20-24). Twelve calves (d 60) from groups A20-35, C20-25, D22-35, and F22-25 (*n* = 3) and another twelve calves (d 180) from groups A18-30, C18-24, D20-30, and F20-24 (*n* = 3) were euthanized. The growth performance parameters were measured. Blood, ruminal fluid, and cecum digesta were collected for further analysis. Results showed heart girth gain of B18-27 was significantly higher than A18-30, C18-24, and heart girth gain (d 180) was significantly affected by protein × starch (DPSRs; *p* < 0.05). Blood urea nitrogen (BUN; d 60) in C20-25 was significantly higher than A20-35 and B20-30 (*p* < 0.05). The BUN (d 180) in D20-30 was significantly higher than A18-30 (*p* < 0.05). The BUN was significantly affected by protein × starch (*p* < 0.05) on d 60. The albumin (ALB) levels in C20-25 and C18-24 were significantly higher than that in A20-35 on d 60 and A18-30 on d 180, respectively (*p* < 0.05). The ALB level in D22-35 on d 60 and E20-27 on d 180 was significantly higher than that in other groups (*p* < 0.05). The ALB level was significantly affected by protein and starch, respectively, on d 60 (*p* < 0.05). In the rumen, the genera *Roseburia* (C20-25) and *Dialister* (D22-35), *Prevotellaceae UCG-001* (C18-24), *Erysipelotrichaceae UCG-002,* and *Anaerovorax* (F20-24) were found in significant higher relative abundances than those in other groups (*p* < 0.05). In the cecum, the genera *Bacteroides* and *Eisenbergiella* (F22-25), *Ruminiclostridium_1* and *Candidatus Stoquefichus* (A18-30), *Erysipelotrichaceae UCG-004* and *Tyzzerella* 4 (D20-30), and *Prevotellaceae UCG-003* and *Klebsiella* (F20-24) were found in significant higher abundances than those in other groups (*p* < 0.05). Collectively, these results indicated that the heart girth, BUN, ALB, and gastrointestinal microbiota responded distinctly to differing DPSRs.

## 1. Introduction

Protein is an essential nutrient for the growth and development of calves. Hence, it is important to accurately determine the protein requirements of dairy calves to be able to provide the appropriate amino acids for rapid structural growth and lean tissue deposition (Blome et al., 2003). However, the optimum protein content of a starter diet for dairy calves remains controversial. The NRC (2001) recommended having 18% crude protein in calf starters for optimum growth. Many studies have been conducted to determine the optimum protein level in calf starter for proper growth and performance. Akayezu et al. (1994) reported that maximum growth was supported by 19.6% protein in the starter and that calf growth did not increase with higher (22.4%) protein content. Hill et al. (2008) suggested that calves should be provided 18% crude protein (CP) in starter up to 8 weeks of age and 15%–16% CP from 8 to 12 weeks. Moreover, starch is considered as the most important energy source required for the growth of calves (Dennis et al., 2018; Aragona et al., 2020). Hence, the starch was used as the main energy source to provide metabolizable energy in the current study. Rauba et al. (2018) indicated that calves that had greater average daily gain (ADG, >0.80 kg/d) consumed more combined protein and metabolizable energy (ME) from milk replacer and starter than calves with lower ADG. Brown et al. (2005) reported that increased energy and protein intake can increase the rate of body growth of heifer calves and potentially reduce rearing costs. Gabler and Heinrichs (2003) reported varying the CP:ME range from 48.3 to 76.5 did not affect the ADG prepubertal Holstein heifers. According to Lammers and Heinrichs (2000), with the ratio of dietary protein to energy increasing, the average daily gain, and feed e fficiency of heifers were increased linearly. So understanding the influence of protein: energy (starch metabolizable energy) on a calf is valuable.

The ruminal microbiota is important for nutrient absorption and energy metabolism of the host (Belanche et al., 2012) and different microbes play different roles in the rumen. For example, bacteria and protozoa break down starch and fiber by releasing cellulolytic enzymes, which are then anaerobically fermented to metabolic intermediates such as volatile fatty acids, ammonia, carbon dioxide, and hydrogen (Kamra, 2005; Kebreab et al., 2009). Ruminants are born without a functional rumen, however, microbial communities exist in a newborn’s rumen, and rumen bacterial communities are thought to be influenced by the diet and age of the animal (Jami et al., 2013; Jewell et al., 2015). Malmuthuge et al. (2013) found that different calf starters affected the bacterial community among gut regions and sample types. In addition, Jami et al. (2013) observed that rumen bacterial diversity and species richness increased with increasing age. Malmuthuge et al. (2014) investigated the composition of the bacteria in the gastrointestinal tracts of pre-weaned bull calves and indicated that the bacterial community was the most abundant in the rumen, followed by the large intestine and then the small intestine.

The relationship between dietary protein and energy to calf growth is well-documented above; however, very limited information exists on how the rumen and the cecum microbiomes are affected by different diet structures during pre-weaning and post-weaning periods. We hypothesized that diet with appropriate crude protein-to-starch metabolizable energy ratios (DPSRs) could improve the growth performance and gastrointestinal microbiota of calves. Therefore, the purpose of this study was to investigate the effects of starters with different DPSRs on the growth performance and rumen and cecum microbiomes of calves.

## 2. Materials and methods

The animals were managed according to the guidelines for the care and use of experimental animals of Jilin Agricultural University Care and Use Committee and commercial dairy farms (JLAU-ACUC2019-018, Changchun, China).

### 2.1. Experimental design

A total of 48 pre-weaning Holstein bull calves with a mean birth weight of 39.75 ± 1.58 kg were selected from a commercial dairy farm, and there was no significant difference in body weight among all groups (*p* > 0.05). As shown in Tables 1, 2, the 48 Holstein bull calves were assigned to six groups (groups A, B, C, D, E, and F) with six dietary DPSRs at d 4 to d 60, and then changed to another six dietary DPSRs at d 61 to d 180. The experiment was designed as a 2 (crude protein, CP) × 3 (starch) randomized complete block. There were 8 pre-weaning calves in groups A20-35 (20% CP and 35% starch), B20-30 (20% CP and 30% starch), C20-25 (20% CP and 25% starch), D22-35 (22% CP and 35% starch), E22-30 (22% CP and 30% starch), and F22-25 (22% CP and 25% starch).

**Table 1:** Ingredients and chemical compositions (% of dry matter) of the experimental diets during the pre-weaning period (4–60 days).

Group^1^	A20-35 (*n* = 8)	B20-30 (*n* = 8)	C20-25 (*n* = 8)	D22-35 (*n* = 8)	E22-30 (*n* = 8)	F22-25 (*n* = 8)
CP	20%			22%		
Starch	35%	30%	25%	35%	30%	25%
**Ingredients**						
Cane molasses	2.00	2.00	2.00	0.40	2.00	2.00
Corn	51.60	41.60	31.50	51.90	42.90	33.00
Soybean meal	28.70	28.20	27.90	37.00	34.20	33.90
Extruded soybean	5.00	5.00	5.00	5.00	5.00	5.00
DDGS	6.00	6.00	6.00	0.00	6.00	6.00
Corn bran	1.00	11.50	21.90	0.00	4.20	14.40
Calcium hydrophosphate	0.30	0.30	0.30	0.30	0.30	0.30
Calcium carbonate	1.50	1.50	1.50	1.50	1.50	1.50
Salt	0.40	0.40	0.40	0.40	0.40	0.40
Baking soda	1.00	1.00	1.00	1.00	1.00	1.00
Premix compound^2^	0.50	0.50	0.50	0.50	0.50	0.50
Yeast culture (Diamond V)	2.00	2.00	2.00	2.00	2.00	2.00
**Chemical compositions**^**3**^						
ME supply by Starch (Mcal/kg)	1.29	1.11	0.92	1.29	1.11	0.92
g of CP/Mcal of Starch ME	155.81	180.18	218.48	170.54	199.10	240.22
NDF/ADF (%)	2.49	2.74	2.88	2.36	2.44	2.66
Ca (%)	0.90	0.90	0.90	0.91	0.91	0.92
ASH (%)	7.63	7.55	7.48	7.61	7.86	7.79

1 The postfix of the groups mean the levels of protein and starch in diet. For example, A20-35 means 20% CP and 35% starch in diet was supplied in group A.

2 Contained 0.19% CuSO_4_∙5H_2_O, 0.39% ZnSO_4_∙H_2_O, 0.28% MnSO4∙H_2_O, 0.12% CoCl_2_, 0.3% Ca(IO_3_)_2_, 0.13% Na_2_SeO_3_, 0.06% vitamin A acetate, 0.01% vitamin D_3_, 0.16% DL-α-tocopherol acetate, 30% CaCO_3_, 20% CaHPO_4_, 0.6% salt, 25% rice hull powder, and 11.36% zeolite.

3 Calculated according to the Feed Composition and Nutritive Values in China (2017).

**Table 2:** Ingredients and chemical compositions (% of dry matter) of the experimental diets during the post-weaning period (61–180 days).

Group^1^	A18-30 (*n* = 5)	B18-27 (*n* = 8)	C18-24 (*n* = 5)	D20-30 (*n* = 5)	E20-27 (*n* = 8)	F20-24 (*n* = 5)
CP	18%			20%		
Starch	30%	27%	24%	30%	27%	24%
**Ingredients**						
Cane molasses	2.00	2.00	2.00	2.00	2.00	2.00
Corn	36.60	30.00	23.30	38.00	31.30	24.80
Extruded soybean	12.90	12.00	11.30	19.00	18.10	17.50
Wheat bran	6.00	8.00	10.00	6.00	8.00	10.00
Cottonseed meal	7.00	7.00	7.00	7.00	7.00	7.00
Corn germ meal	7.00	7.00	7.00	7.00	7.00	7.00
DDGS	12.00	12.00	12.00	12.00	12.00	12.00
Corn bran	13.60	19.10	24.50	6.10	11.70	16.80
Calcium hydrophosphate	0.50	0.50	0.50	0.50	0.50	0.50
Calcium carbonate	1.40	1.40	1.40	1.40	1.40	1.40
Salt	0.50	0.50	0.50	0.50	0.50	0.50
Premix compound	0.50	0.50	0.50	0.50	0.50	0.50
**Chemical compositions**^**2**^						
ME supply by Starch (Mcal/kg)	1.10	0.99	0.88	1.10	0.99	0.88
g of CP/Mcal of Starch ME	167.00	185.05	208.52	184.91	205.05	231.25
NDF/ADF(%)	2.91	2.97	3.01	2.71	2.80	2.86
Ca (%)	0.90	0.90	0.90	0.91	0.91	0.92
ASH (%)	7.08	7.08	7.10	7.37	7.37	7.39

1 The postfix of the groups mean the levels of protein and starch in diet. For example, A18-30 means 18% CP and 30% starch in diet was supplied in group A.

2 Calculated according to the Feed Composition and Nutritive Values in China (2017).

### 2.2. Animal housing and feeding

The calves during the pre-weaning period were housed in individual calf hutches (2.20 m × 1.32 m × 1.39 m) with pens (1.75 m × 1.30 m × 1.10 m) until d 60. The calf hutches were bedded with sand and pens were equipped with a trough (diameter: 25 cm, depth: 16 cm) for the calf starter and a bucket for milk and water. The calves were given colostrum (IgG > 50 mg/mL) at 5 L/d for 4 days and the calf starter was offered from d 4. The calves were fed up to 6 L/d of commercial milk replacer (Sprayfo Red, Sloten BV, Antwerpenweg 7, 7418 CR Deventer, Netherlands) from d 5 to d 7. The amount of milk replacer fed to calves was gradually increased to 7 L/d and 8 L/d from d 8 to d 14 and d 15 to d 21, respectively. From d 22 to d 37 and d 38 to d 51, calves were fed milk replacer at 9 and 10 L/d, respectively. On day 52, weaning was initiated by gradually reducing the milk replacer to 1 L/d. So, the calves were completely weaned off milk replacer at d 60. Each calf drank milk twice a day during the pre-weaning period. At d 61, the calves were not fed milk replacer and moved to 6 pens (6.2 m × 5.0 m) according to their treatments. The d 61 to d 66 constituted the transition period to using the two-stage diets according to the management system; the calf starter was gradually decreased and the concentrate (post-weaning period) was gradually increased in different ratios (proportion of starter to concentrate: 6:1, 5:2, 4:3, 3:4, 2:5, and 1:6 on d 61, d 62, d 63, d 64, d 65, and d 66, respectively). Each post-weaning calf began to feed on oat grass (0.2 kg/d) at d 90 and the calves were fed twice daily. Water, calf starter, and concentrate were always freely available.

### 2.3. Sample’s collection and analysis

In consideration of animal ethics, not all calves of all groups were killed. Twelve calves at age of d 60 from groups A20-35 (*n* = 3), C20-25 (*n* = 3), D22-35 (*n* = 3), and F22-25 (*n* = 3) were euthanized. There were 8 post-weaning calves in groups B18-27 (18% CP and 27% starch) and E20-27 (20% CP and 27% starch), and 5 post-weaning calves in groups A18-30 (18% CP and 30% starch), C18-24 (18% CP and 24% starch), D20-30 (20% CP and 30% starch), and F20-24 (20% CP and 24% starch). Twelve calves from groups A18-30 (*n* = 3), C18-24 (*n* = 3), D20-30 (*n* = 3), and F20-24 (*n* = 3) at age of d 180 were euthanized. The ruminal fluid of these dead animals was collected from three different positions (top, middle, and bottom) and was mixed, and the cecum digesta were collected at 5 cm from the junction of the ileum and cecum on d 60 and d 180. The sample collection process of ruminal fluid and cecum digesta was conducted by one person to reduce experimental error, respectively. Then sampled and immediately frozen in liquid nitrogen and stored at −40°C for microbiome analysis by high-throughput sequencing. Body weight, withers height, body length, heart girth, and cannon bone circumference were measured on d 4, 60, and 180. Blood was sampled *via* the jugular vein with 10 mL vacuum blood collection tubes and needle (0.70 mm × 25 mm) before morning feeding on d 60 and 180, and serum total protein (TP), albumin (ALB), and blood urea nitrogen (BUN) were analyzed using an automatic biochemical analyzer (Indiko 863, Thermo Fisher Scientific Oy, Massachusetts, United States).

### 2.4. DNA extraction and sequencing

Total genomic DNA samples were extracted using Fast DNA SPIN extraction kits (MP Biotechnology, CA, United States) according to the manufacturer’s specifications. The DNA concentration was measured using a NanoDrop ND-1000 (Thermo Scientific, Massachusetts, United States). The V3–V4 regions of the 16S rRNA genes were amplified using the universal primers 338F (5′-ACTCCTACGGGAGGCAGCA-3′) and 806R (5′-GGACTACHVGGGTWTCTAAT-3′). The PCR was conducted according to a previous method (Dill-McFarland et al., 2017). The PCR products were purified and quantified using VAHTSTM DNA Clean Beads (Vazyme, Nanjing, China) and PicoGreen dsDNA Assay Kit (Invitrogen, Carlsbad, CA, United States), respectively. The purified PCR products were pooled and sequenced using the Illumina MiSeq platform with a MiSeq Reagent Kit v3 at Shanghai Personal Biotechnology Co., Ltd. (Shanghai, China). The sequencing data were processed using the Quantitative Insights into Microbial Ecology (QIIME 2) pipeline (Bolyen et al., 2018). Briefly, the primer sequence was trimmed off and the remaining sequence was then quality filtered, denoised, and merged, and chimeras were removed using the DADA2 plugin (Callahan et al., 2016). The remaining high-quality sequences were re-clustered at 97% to generate amplicon sequence variants (ASVs) using the clustering program Vsearch (v2.13.4; Rognes et al., 2016). The sample sequence was flattened based on ASV, and the average score of the maximum flattening depth was selected to calculate the Alpha diversity indices such as Chao1, Shannon, and Pielou_e. The amplicon sequence data were deposited with the National Center for Biotechnology Information (Accession No. SRP348482).

### 2.5. Statistical analyses

Data were analyzed by the SPSS software version 23.0, the data were analyzed as a linear mixed-effects model, which included crude protein, starch (starch metabolizable energy), and their interaction (CP × starch; DPSRs) as fixed effects. Data were analyzed using the model: Y*_ijk_* = μ + F*_i_* + V*_j_* + F*_i_* × V*_j_* + e*_ijk_*, where μ is the overall mean, F*_i_* is the effect of crude protein (*i* = 1–2), V*_j_* is the effect of starch (*j* = 1–3), F*_i_* × V*_j_* is the dietary crude protein × starch level interaction, and e*_ijk_* is the residual effect. Then, the treatment groups of pre-weaning and post-weaning period were analyzed by one-way ANOVA, respectively. The results were expressed as the mean ± SE (standard error), and the differences among means were tested for significance by Duncan’s multiple range test. *p* < 0.05 was considered to indicate a statistically significant difference and tendency toward significance was declared at 0.05 < *p* ≤ 0.10.

## 3. Results

### 3.1. Growth performance

No significant difference was observed in overall growth performance during the experimental period (*p* > 0.05, Table 3). For lower protein level (pre-weaning period: 20%, post-weaning: 18%) treatments, the average body weight on days 60 and 180, and ADG from d 4 to d 60 of group C20-25 were higher than other groups, whereas, ADG of group B18-27 was highest from d 61 to d 180 (*p* > 0.05). For higher protein level (pre-weaning period: 22%, post-weaning period: 20%) treatments, average body weight on d 60 and ADG of group D22-35 were higher than other groups before weaning; however, average body weight on d 180 and ADG of group E20-27 were higher than other groups after weaning (*p* > 0.05). We observed a significant protein × starch interaction (DPSRs) for heart girth (*p* = 0.002) on d 180. Heart girth gain of group B18-27 was significantly higher than groups A18-30 and C18-24 on d 180 (*p* < 0.05). When the starch level in the diet was higher, the body length gain and cannon bone circumference gain of group A18-30 were significantly higher than for group D20-30 (*p* < 0.05). Cannon bone circumference gain was significantly affected independently by protein (*p* = 0.02) and starch (*p* = 0.01) on d 180. Therefore, according to these data, the calves fed the 20% CP in the starter with a ratio of 155.81 g (group A20-35) and 218.28 g (group C20-25) of CP/Mcal of starch and 22% CP in the starter with a ratio of 150.54 g (group D22-35) and 240.22 g (group F22-25) of CP/Mcal of starch were euthanized on d 61 for further research.

**Table 3:** Growth performance and body measurements during the experimental period.

Pre-weaning (d 4–60)							*p*-value		
Item	A20-35^1^ (*n* = 8)	B20-30 (*n* = 8)	C20-25 (*n* = 8)	D22-35 (*n* = 8)	E22-30 (*n* = 8)	F22-25 (*n* = 8)	CP	Starch	CP × starch (DPSRs)
Body weight (d 4), kg	39.75 ± 0.59	39.57 ± 0.65	39.57 ± 0.65	39.75 ± 0.59	39.43 ± 0.57	39.43 ± 0.68	0.85	0.89	0.99
Body weight (d 60), kg	79.25 ± 3.42	81.77 ± 3.86	88.89 ± 2.83	86.37 ± 2.89	81.70 ± 3.51	83.78 ± 2.71	0.36	0.12	0.91
Average daily gain, kg/d	0.71 ± 0.06	0.75 ± 0.07	0.88 ± 0.05	0.83 ± 0.05	0.75 ± 0.07	0.79 ± 0.05	0.12	0.40	0.89
Withers height gain, cm/d	0.21 ± 0.01	0.19 ± 0.02	0.19 ± 0.01	0.20 ± 0.03	0.23 ± 0.02	0.25 ± 0.03	0.09	0.88	0.20
Heart girth gain, cm/d	0.37 ± 0.02	0.34 ± 0.02	0.39 ± 0.02	0.39 ± 0.02	0.36 ± 0.03	0.42 ± 0.03	0.33	0.09	0.96
Body length gain, cm/d	0.32 ± 0.02	0.36 ± 0.05	0.37 ± 0.03	0.27 ± 0.03	0.36 ± 0.03	0.35 ± 0.03	0.43	0.08	0.71
Cannon bone circumference gain, cm/d	0.03 ± 0.00	0.03 ± 0.01	0.02 ± 0.01	0.02 ± 0.00	0.02 ± 0.01	0.03 ± 0.00	0.44	0.91	0.52
Post-weaning period (d 61–180)							*p*-value		
Item	A18-30 (*n* = 5)	B18-27 (*n* = 8)	C18-24 (*n* = 5)	D20-30 (*n* = 5)	E20-27 (*n* = 8)	F20-24 (*n* = 5)	CP	Starch	CP × starch (DPSRs)
Body weight (d 180), kg	224.62 ± 4.47	244.26 ± 8.71	246.84 ± 15.03	223.66 ± 15.35	229.96 ± 7.06	223.94 ± 13.04	0.43	0.46	0.81
Average daily gain, kg/d	1.20 ± 0.04	1.32 ± 0.06	1.26 ± 0.09	1.13 ± 0.10	1.24 ± 0.04	1.18 ± 0.09	0.51	0.22	0.66
Withers height gain, cm/d	0.12 ± 0.01	0.12 ± 0.01	0.11 ± 0.01	0.11 ± 0.04	0.12 ± 0.01	0.14 ± 0.02	0.42	0.86	0.33
Heart girth gain, cm/d	0.18 ± 0.02^ab^	0.26 ± 0.02^c^	0.20 ± 0.02^abc^	0.23 ± 0.03^bc^	0.16 ± 0.01^a^	0.20 ± 0.02^abc^	0.35	0.60	0.002
Body length gain, cm/d	0.30 ± 0.01^b^	0.28 ± 0.01^ab^	0.29 ± 0.03^b^	0.22 ± 0.03^a^	0.28 ± 0.01^ab^	0.24 ± 0.02^ab^	0.28	0.55	0.43
cannon bone circumference gain, cm/d	0.06 ± 0.00^bc^	0.06 ± 0.00^bc^	0.04 ± 0.00^a^	0.04 ± 0.00^a^	0.05 ± 0.00^ab^	0.05 ± 0.01^ab^	0.02	0.01	0.05

a, b, c means in the same row followed by different superscripts differ at *p* < 0.05.

1 The postfix of the groups mean the levels of protein and starch in diet. For example, A20-35 means 20% CP and 35% starch in diet was supplied in group A.

### 3.2. Blood index

The total protein (TP) levels in serum were not significantly different among the groups before weaning when calves were fed different diets (Table 4). When the protein amount in the diet was lower (18% or 20%), the ALB level increased with DPSRs increasing during the experimental period, and the ALB level in group C20-25 and C18-24 was significantly higher than that in group A20-35 on d 60 and group A18-30 on d 180, respectively (*p* < 0.05). ALB level was significantly affected independently by protein and starch, respectively, on d 60 (*p* < 0.05). For groups with a higher CP level, the ALB level in the group D22-35 on d 60 and group E20-27 on d 180 was significantly higher than that in other groups (*p* < 0.05). For the groups with the same starch level, the urea amount was significantly affected by starch and protein × starch on d 60 (*p* < 0.05) and increased with higher protein level compared to the urea amount in groups with a lower protein level on d 180.

**Table 4:** Effect of dietary protein-to-starch metabolizable energy ratios on blood index.

d 60							*p*-value		
Item	A20-35 (*n* = 8)	B20-30 (*n* = 8)	C20-25 (*n* = 8)	D22-35 (*n* = 8)	E22-30 (*n* = 8)	F22-25 (*n* = 8)	CP	Starch	CP × starch (DPSRs)
Total protein (TP; g/L)	61.08 ± 2.00	61.07 ± 1.91	59.41 ± 1.38	59.23 ± 1.30	58.80 ± 1.96	56.72 ± 1.31	0.19	0.26	0.76
Albumin (ALB; g/L)	31.34 ± 0.44 ^a^	31.27 ± 0.99 ^a^	34.14 ± 0.69 ^bc^	35.42 ± 0.72 ^c^	32.67 ± 1.09 ^ab^	34.61 ± 0.42 ^bc^	0.00	0.02	0.07
Urea (mmol/L)	3.34 ± 0.16 ^a^	3.32 ± 0.30 ^a^	4.86 ± 0.30 ^c^	4.43 ± 0.42 ^bc^	3.64 ± 0.31 ^ab^	4.05 ± 0.24 ^abc^	0.84	0.04	0.03
d 180							*p*-value		
Item	A18-30 (*n* = 5)	B18-27 (*n* = 8)	C18-24 (*n* = 5)	D20-30 (*n* = 5)	E20-27 (*n* = 8)	F20-24 (*n* = 5)	CP	Starch	CP × starch (DPSRs)
Total protein (TP; g/L)	62.95 ± 1.72	64.12 ± 1.18	63.37 ± 1.60	64.48 ± 1.61	64.10 ± 1.02	65.90 ± 1.91	0.16	0.93	0.41
Albumin (ALB; g/L)	32.56 ± 1.41 ^a^	35.56 ± 0.46 ^ab^	36.16 ± 0.72 ^b^	34.50 ± 1.51 ^ab^	35.57 ± 0.33 ^ab^	35.08 ± 1.50 ^ab^	0.85	0.21	0.79
Urea (mmol/L)	4.89 ± 0.19 ^a^	5.11 ± 0.11 ^ab^	4.80 ± 0.41 ^a^	5.65 ± 0.25 ^b^	5.28 ± 0.13 ^ab^	5.00 ± 0.32 ^ab^	0.10	0.09	0.53

a, b, c means in the same row followed by different superscripts differ at *p* < 0.05.

1 The postfix of the groups mean the levels of protein and starch in diet. For example, A20-35 means 20% CP and 35% starch in diet was supplied in group A.

### 3.3. Composition of rumen and cecum microbiomes

**Figure 1:**
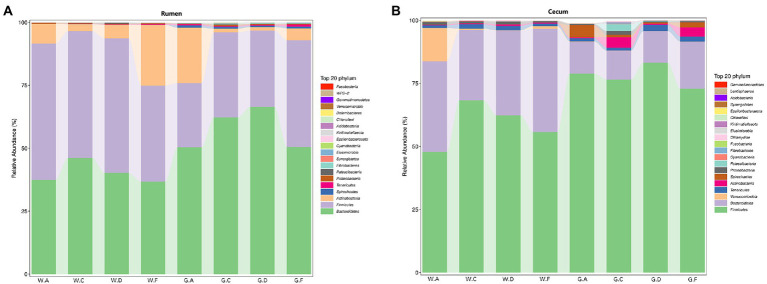
Relative abundance of microbial communities in calves at the phylum level: **(A)** rumen; **(B)** cecum; The postfix of the groups mean the levels of protein and starch in diet: A20-35, B20-30, C20-25, D22-35, E22-30, F22-25; A18-30, B18-27, C18-24, D20-30, E20-27, and F20-24. For example, A20-35 means 20% CP and 35% starch in diet was supplied in group A.

At the phylum level, the rumens of different groups had similar dominant microbial communities, which included Firmicutes, Bacteroidetes, and Actinobacteria (Figure 1A). However, the abundance of the same phyla in the pre-weaning and post-weaning periods showed differences. For instance, Firmicutes was the predominant phylum, followed by Bacteroidetes, in the rumen during the pre-weaning period, but the opposite result was detected in groups during the post-weaning period, during which Bacteroidetes was the predominant phylum. The abundance of Bacteroidetes in the rumen of groups C20-25 and D20-30 was higher than that in other groups in pre-weaning and post-weaning periods. However, Bacteroidetes was more abundant in the cecum of groups F22-25 (F20-24) than that in other groups in pre-weaning and post-weaning periods (Figures 1A,B). Visible differences in the microbial relative abundance were detected between the rumen and the cecum. Firmicutes, Bacteroidetes, Tenericutes, and Verrucomicrobia were the four most dominant phyla in the cecum (Figure 1B). In both the pre-weaning and post-weaning periods, the abundance of Firmicutes in the cecum was higher than that of Bacteroidetes, and the abundance of Firmicutes groups C20-25 and G20-30 was more abundant than that in other groups in pre-weaning and post-weaning periods.

**Figure 2:**
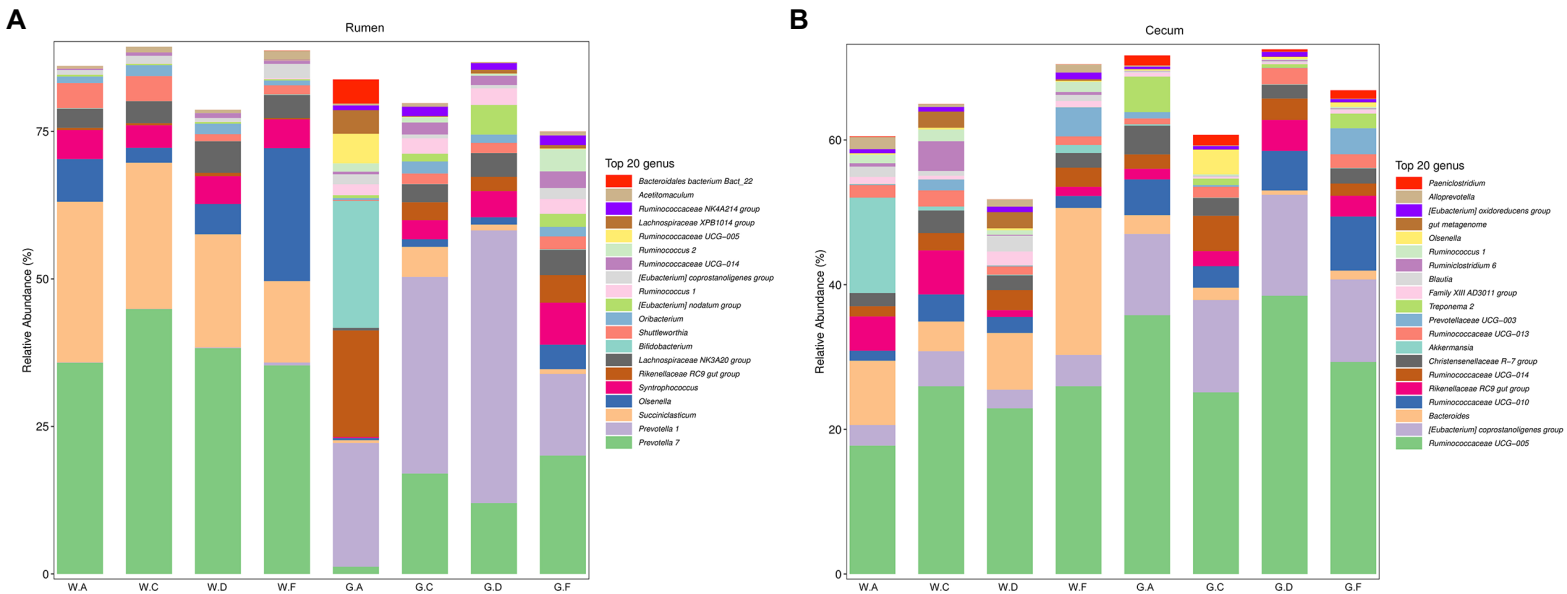
Relative abundance of microbial communities in calves at the genus level: **(A)** rumen; **(B)** cecum; The postfix of the groups mean the levels of protein and starch in diet: A20-35, B20-30, C20-25, D22-35, E22-30, F22-25; A18-30, B18-27, C18-24, D20-30, E20-27, and F20-24. For example, A20-35 means 20% CP and 35% starch in diet was supplied in group A.

At the genus level, 284 genera in the rumen and 381 genera in the cecum were found in the weaning period. During the post-weaning period, 388 and 454 genera were detected in the rumen and cecum, respectively. Additionally, most genera in both the rumen and cecum belonged to Firmicutes and Bacteroidetes. Figures 2A,B show the genera whose relative abundance was more than 1%. In the rumen, *Prevotella 7* was the most abundant genus during the pre-weaning period (average relative abundance more than 35% in different groups), whereas *Prevotella 1* in groups C18-24 (average relative abundance was 33%) and D20-30 (average relative abundance was 46%), the genus *Bifidobacterium* in group A18-30 (average relative abundance was 22%), and the genus *Prevotella 7* in group F20-24 (average relative abundance was 20%) were prominent during the post-weaning period, respectively. The abundance of *Succiniclasticum* was higher during the pre-weaning period than in the post-weaning period. In the cecum, the relative abundance of *Ruminococcaceae UCG-005* was the highest (average relative abundance was from 18 to 38%) during the pre-weaning and post-weaning periods. Genus *Ruminococcaceae UCG-005* was more abundant in high DPSRs groups (groups C20-25 and F22-25) than that in low DPSRs groups (groups A20-35 and D22-35) on d 60; however, opposite results were observed on d 180, which was *Ruminococcaceae* UCG-005 was more abundant in low DPSRs groups (groups A18-30 and D20-30) than that in high DPSRs groups (groups C18-24 and F20-24) on d 180. Bacteroides and [Eubacterium]coprostanoligenes were the second most abundant genera in the pre-weaning and post-weaning periods, respectively.

**Figure 3:**
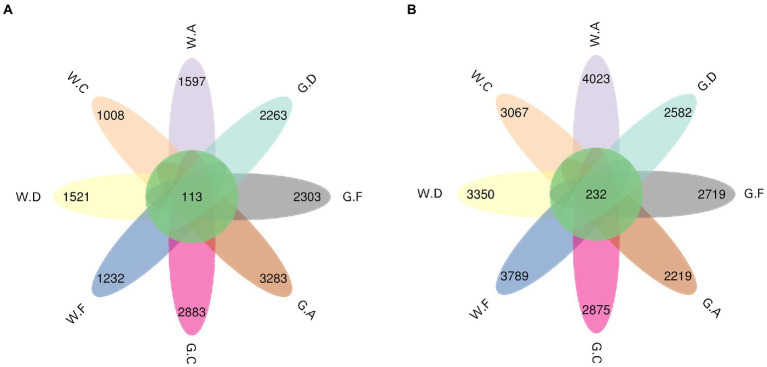
ASV flower petal map of the taxa: **(A)** rumen; **(B)** cecum; The postfix of the groups mean the levels of protein and starch in diet: A20-35, B20-30, C20-25, D22-35, E22-30, F22-25; A18-30, B18-27, C18-24, D20-30, E20-27, and F20-24. For example, A20-35 means 20% CP and 35% starch in diet was supplied in group A.

**Figure 4:**
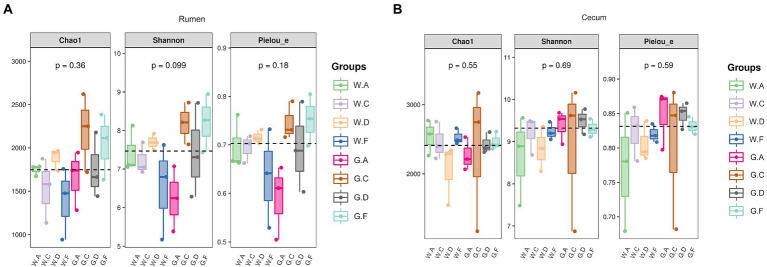
Alpha diversity index: **(A)** rumen; **(B)** cecum; The postfix of the groups mean the levels of protein and starch in diet: A20-35, B20-30, C20-25, D22-35, E22-30, F22-25; A18-30, B18-27, C18-24, D20-30, E20-27, and F20-24. For example, A20-35 means 20% CP and 35% starch in diet was supplied in group A.

The result showed that the ASV flower petal map of rumen microbes was increased during the post-weaning period when compared with the pre-weaning period; however, the opposite results were found in the cecum (Figures 3A,B). The number of ASV in the rumen of group A18-30 was higher than that of other groups during the post-weaning period. The number of ASV in the cecum of group A20-35 was the highest and that of group C20-25 was the lowest on d 60; however, the opposite results of ASV were shown on d 180, which group C18-24 was the highest and group A18-30 was the lowest. No significant difference in Alpha diversity indexes about rumen and cecum was obtained in the present research, however, the variation trend of microbes could be observed (Figures 4A,B). For the lower protein groups (pre-weaning period: 20% CP; post-weaning period: 18% CP), Chao1, Shannon, and Pielou_e in the rumen of group A20-35 were higher than those of other groups (*p* > 0.05) during the pre-weaning period, and Chao1, Shannon, and Pielou_e of group D20-30 were higher than those of other groups (*p* > 0.05) during the post-weaning period. Chao1 in the cecum of group A20-35 and Shannon and Pielou of group C20-25were higher than those of other groups (*p* > 0.05) during the pre-weaning period. Chao1 in the cecum of group C18-24, and Shannon and Pielou of group A18-30 were higher than those of other groups (*p* > 0.05) during the post-weaning period. For the higher protein groups (pre-weaning period: 22% CP; post-weaning period: 20% CP), Chao1, Shannon, and Pielou_e in the rumen of group C20-25 were higher than those of other groups (*p* > 0.05) during the pre-weaning period, and Chao1, Shannon, and Pielou_e of group F20-24 were higher than those of other groups (*p* > 0.05) during the post-weaning period. Chao1, Shannon, and Pielou_e in the cecum of group F22-25 were higher than those of other groups (*p* > 0.05) during the pre-weaning period, and Chao1of group F20-24 and Shannon and Pielou_e of group D20-30 were higher than those of other groups (*p* > 0.05).

**Figure 5:**
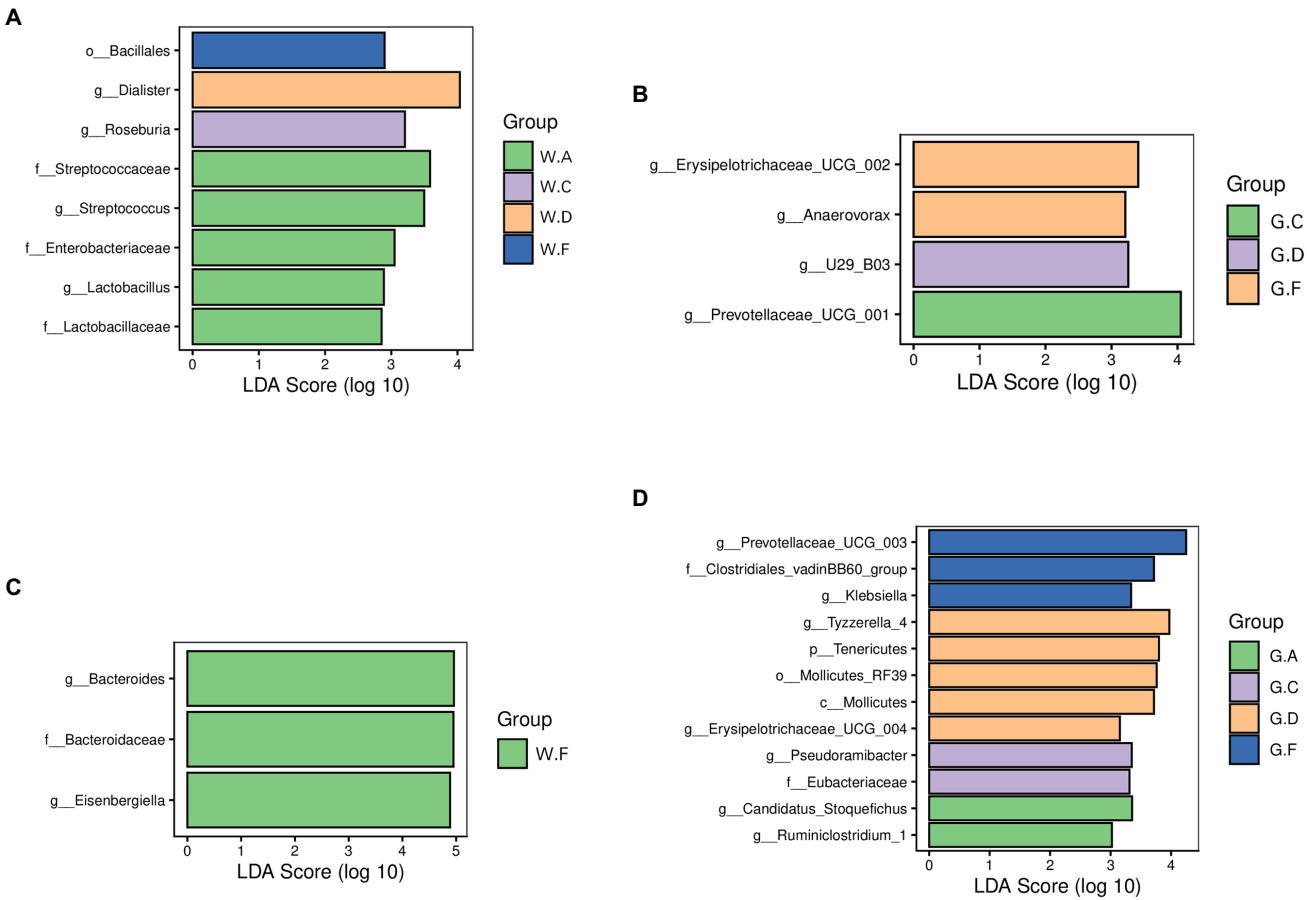
Differences in the microbial communities of different groups: **(A,B)** rumen; **(C,D)** cecum; The postfix of the groups mean the levels of protein and starch in diet: A20-35, B20-30, C20-25, D22-35, E22-30, F22-25; A18-30, B18-27, C18-24, D20-30, E20-27, and F20-24. For example, A20-35 means 20% CP and 35% starch in diet was supplied in group A.

To identify specific microbial candidates present in the different groups during the pre-weaning and post-weaning periods, LEFSE (LDA Score > 2) and *p* < 0.05 were used to determine biomarkers belonging to different taxa. In the rumen, the families Lactobacillaceae, Streptococcaceae, and Enterobacteriaceae were higher in the group A20-35 during the pre-weaning period. The genera *Roseburia* and *Prevotellaceae UCG-001* were higher in groups C20-25 and C18-24 during the pre-weaning and post-weaning periods, respectively. The genera *Dialister* and *U29-B03* were higher in groups D22-35 and D20-30 during the pre-weaning and post-weaning periods, respectively. The order Bacillales was the biomarker in group F22-25 during the pre-weaning period, and the genera *Erysipelotrichaceae UCG-002* and *Anaerovorax* in group F20-24 were found in higher abundances during the post-weaning period (Figures 5A,B). In the cecum, the family Bacteroidaceae and the genus *Bacteroides* and *Eisenbergiella* were the biomarkers in group F22-25 during the pre-weaning period. The genera *Ruminiclostridium_1* and *Candidatus Stoquefichus* in group A18-30; the family Eubacteriaceae in group C18-24; the phylum Tenericutes and the genera *Erysipelotrichaceae UCG-004* and *Tyzzerella 4* in group D20-30; and the family Clostridiales vadin BB60 and the genera *Prevotellaceae UCG-003* and *Klebsiella* in group F20-24 during the post-weaning were found in higher abundances (Figures 5C,D).

### 3.4. Network analysis of microbial metabolic pathways

**Figure 6:**
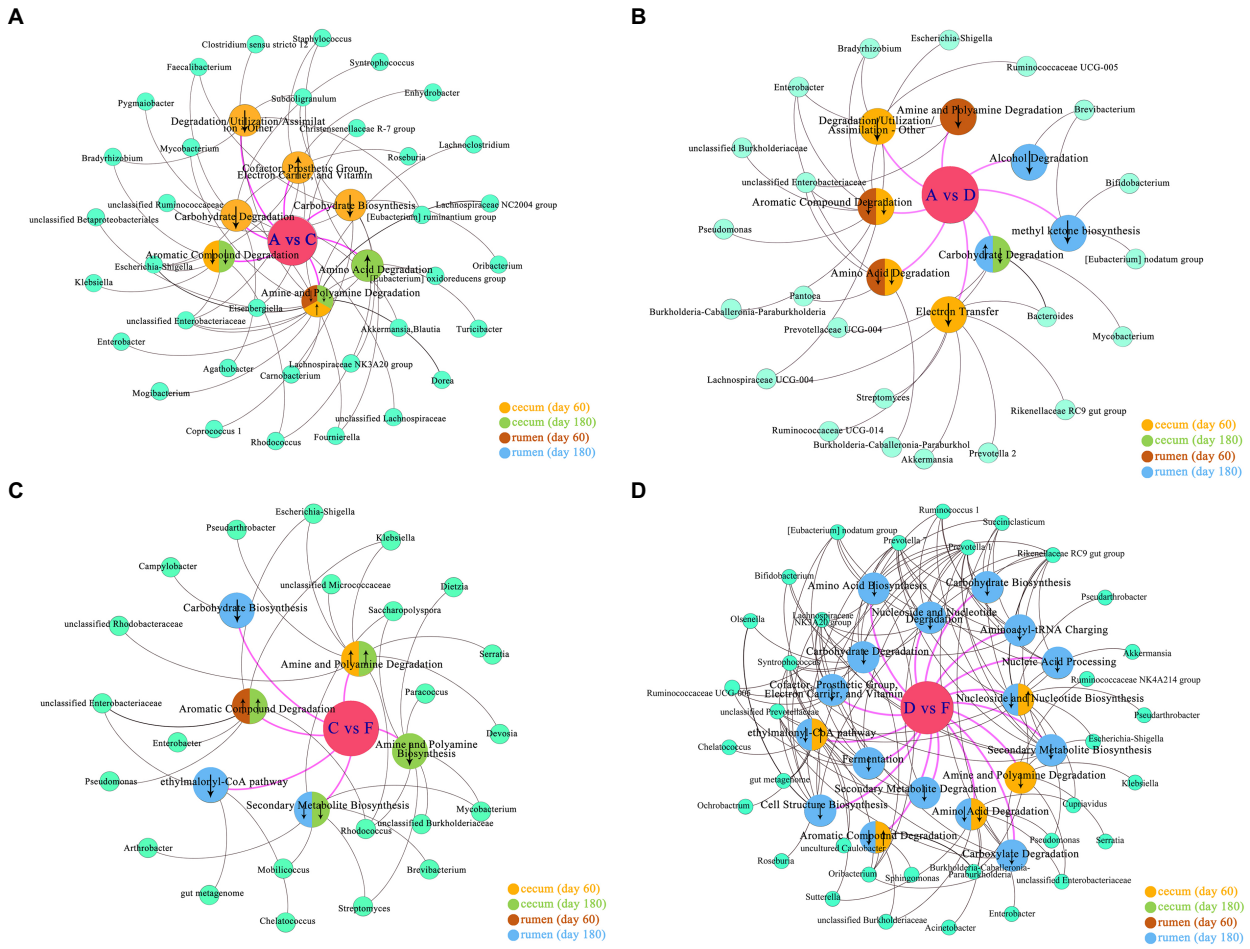
Predicted pathway and network analysis of microbiome: **(A)** group A (A20-35, A18-30) vs. group C (C20-25, C18-24); **(B)** group A (A20-35, A18-30) vs. group D (D22-35, D20-30); **(C)** group C (C20-25, C18-24) vs. group F (F22-25, F20-24); **(D)** group D (D22-35, D20-30) vs. group F (F22-25, F20-24). The biggest circles in the middle position, such as A vs. C: the metabolic pathway changes of group C compared with group A. The second biggest circles represent pathways related to the rumen or cecum. The smallest circles represent the microbes involved in the pathways. ↑ indicates metabolite pathway was promoted; ↓ indicates metabolite pathway was downregulated.

Microbiomes of different groups were imported into the MetaCyc database^1^ for pathway analysis, and the significant metabolic pathways (*p* < 0.05) with microbes are shown in Figure 6 when compared among different levels of DPSRs. A total of 6, 18, 10, and 6 pathways were related to microbiomes in the rumen (d 60, d 180) and the cecum (d 60, d 180), respectively. In the lower protein groups (pre-weaning period: 20% CP; post-weaning period, 18% CP; Figure 6A, A vs. C, Figure 6B, A vs. D), microbiomes in the rumen (d 60) and cecum (d 60, d 180) were involved in the common pathways of aromatic compound degradation, amine and polyamine degradation, and amino acid degradation, and the rumen (d 180) and cecum (d 60, d 180) were involved in the common pathway of carbohydrate degradation. For lower CP groups, the pathways of the cofactor, prosthetic group, electron carrier, vitamin biosynthesis(group C20-25), amine and polyamine degradation (group C20-25), and amino acid degradation (group C18-24) were promoted in the cecum in high DPSRs groups compared with low DPSRs group; however, other related pathways in the rumen and cecum were restrained. For the higher CP groups (pre-weaning period: 22% CP; post-weaning period: 20% CP; Figure 6D, D vs. F), the pathways of aromatic compound degradation (group F22-25), ethylmalonyl-CoA pathway (group F22-25), and nucleoside and nucleotide biosynthesis (group F22-25) were promoted in the cecum in higher DPSRs group when compared with the lower DPSRs group, whereas other related pathways in the rumen and the cecum were restrained. For the 35% starch level groups (Figure 6B, A vs. D), only the pathway of carbohydrate degradation, related to the genus Bacteroides in the rumen on d 180, was promoted with increasing CP. For the 25% starch level groups (Figure 6C, C vs. F), the pathways of amine and polyamine degradation (groups F22-25 and F20-24) in the cecum, and aromatic compound degradation (groups F20-24) in cecum and rumen were promoted in the 22% CP groups when compared with 20% CP groups.

## 4. Discussion

### 4.1. Growth performance

The proper nutrition level and diet structure are critical for the growth and development of calves, especially the level of dietary protein and energy, and the ratio of protein to energy. Zothanpuii et al. (2015) found that crossbred calves fed lower protein calf starter with higher nitrogen-free extract (22% CP) showed a significantly increased body weight compared to those fed calf starters with higher protein (24% and 26% CP) and lower nitrogen-free extract. Our results showed the similar growth tendency; however, no significant difference among different groups, which we hypothesized the BW appears to be affected by many factors, including the individual difference and interaction of the level of protein and starch in the diet. Stamey et al. (2012) found that a high-CP starter (25.5% CP; ME: 3.21 Mcal/kg) could significantly increase the ADG of calves compared to those fed conventional starter (19.6% CP, ME: 3.16 Mcal/kg) before weaning. Yousefinejad et al. (2021) reported that female Holstein dairy calves supplied with 22% CP in starter showed greater overall ADG, pre-weaning BW, and feed efficiency than that supplied with 18% CP. In the present study, all the calves were fed the same amount of milk before weaning, and it should be noted that, when the diet with lower CP (20% CP in the pre-weaning period and 18% CP in the post-weaning periods) was provided to the calves during the experimental period, their growth was not constrained during the developmental stage, i.e., when their requirements for protein were high. This is probably because the digestible energy intake that was provided by starch was sufficient. For the groups with the same level of protein, lower CP (20%) with lower starch (25%) of 218.48 g of CP/Mcal of starch ME and higher CP (22%) with higher starch (35%; 170.54 g of CP/Mcal of Starch ME) showed the higher BW during the pre-weaning period. Similar results were found by Lammers and Heinrichs (2000), which found that calf starters with 35.3%, 33.4%, and 31.4% starch and 27.1%, 25%, and 24.7% CP, respectively, did not significantly affect ADG. However, in the current study, higher CP (20%) with higher starch (35%) did not affect the body weight of calves when compared with the lower levels of starch (30 and 25%) during the post-weaning period. Accordingly, different levels of CP could influence calf performance, but a proper combination of dietary nutrient levels and CP: ME ratio balance for calves at different stages of development may reduce or eliminate this effect and may even benefit calf performance. Lower CP (20%) with lower starch (25%; 218.48 g of CP/Mcal of Starch ME) or higher CP with higher starch (170.54 of CP/Mcal of Starch ME) before weaning, and lower CP with medium-level starch (180.05 of CP/Mcal of Starch ME) or higher CP with medium-level starch (205.05 of CP/Mcal of Starch ME) showed greater benefits for calf performance.

### 4.2. Blood index

The serum TP did not change considerably with different DPSRs, but the ALB concentrations in the groups C20-25 and C18-24 (higher DPSRs) was higher than that in groups A20-35 (A18-30) and B20-30 (B18-27), and the group D22-35 (lower DPSRs) were higher than that in groups E22-30 and F22-25 in the current study. In accordance with the present results, Daneshvar et al. (2016) and Makizadeh et al. (2020) reported no significant change in TP after feeding calves with different levels of CP; furthermore, both results showed no significant effect on ALB concentration. In contrast to our results, Sharma et al. (2019) found that TP concentration increased with higher levels of protein in the starter, but no significant change was observed in ALB. Blood urea N (BUN) concentration is generally considered an indicator of protein status (Kohn et al., 2005). In the present study, the BUN concentration was higher in the 20% CP groups than in the 18% CP groups during the post-weaning period, which may be due to excess ruminal nitrogen concentrations and lower protein requirements for the growth than in the pre-weaning period (Kazemi-Bonchenari et al., 2018). For the 20% CP groups, the concentration of BUN was decreasing with the starch decreasing; however, seldom research was about the effect of starch on the blood index. We hypothesized that the changes in blood might be related to growth performance and the ADG was lowest when the level of starch was 27% in the current study.

### 4.3. Composition of rumen and cecum microbiomes

The current study characterized the ruminal and cecal bacterial composition in calves with different DPSRs in the feeding trial; we also identified variations in bacterial diversity and specific ruminal and cecal bacteria in different groups. Jami et al. (2013) found that the ruminal bacterial diversity of calves increased from 2 to 6 months of age, which is in agreement with the current results. The ruminal bacterial community during the post-weaning period was more abundant than in the pre-weaning period; however, the opposite trend was found in the cecal bacterial community, probably because the calf rumen is not fully matured by d 60 its function was similar as monogastric animals. Thus, the cecal bacterial diversity was greater than that of the rumen, which was consistent with the results of Malmuthuge et al. (2014). During the post-weaning (d 180), calves developed a more mature rumen, suggesting a more diverse but specific and homogeneous bacterial community (Jami et al., 2013). In the present study, the same dominant phyla were present in the rumen and cecum, although the relative abundances varied depending on the calf starter and the stage of development. Moreover, the relative abundance of Bacteroidetes increased, and that of Firmicutes decreased in the rumen from pre-weaning to post-weaning, which is inconsistent with the findings of Jami et al. (2013) and Meale et al. (2016). Conversely, the relative abundance of Bacteroidetes decreased and that of Firmicutes increased in the cecum from d 60 to d 180. *Prevotella* belongs to the phylum Bacteroidetes, and *Prevotella* can produce a range of xylanases (Krause et al., 2003) and may contribute to the efficient utilization of hemicellulose pectin and protein (Osborne and Dehority, 1989; Satoshi and Yasuo, 2001). The increase in Bacteroidetes in the rumen is indicative of the gradual rumen development to maturity and is promoted by processes utilizing oat grass and calf starter during the post-weaning period.

Previous studies have reported that diet is one of the main factors that can change gut microbial diversity (Ley et al., 2008; Maslowski and Mackay, 2011; Dill-Mcfarland et al., 2019). The abundance of each phylum or genus was influenced when calves were supplied with different DPSRs in the present study. In agreement with a previous study in dairy calves by Meale et al. (2016), the genus *Succiniclasticum* was more abundant in pre-weaning than that in post-weaning. Succiniclasticum specializes in fermenting succinate, quantitatively converting it to propionate (Gylswyk, 1995); furthermore, a higher proportion of propionate will be produced in the rumen compared with the period of supplementation with oat grass after weaning (Kim et al., 2016). In this case, the currently observed decrease in abundance of *Succiniclasticum* may have been because no forage was supplemented for calves before weaning. Petri et al. (2013) reported that *Succiniclasticum* in heifers fed a high-grain diet was more abundant than in those fed forages or a mixed forage diet. Moreover, the current study showed that the phylum Bacteroidetes was dominated by the genus *Prevotella* in the rumen at all stages of development; however, in the cecum, the genus *Bacteroides* was the most abundant, although the abundance decreased after weaning, which was also found by Meale et al. (2016). Furthermore, for groups with the same level of CP, most of the pathways in which microbes in the rumen and cecum are involved became restrained when the starch concentration in the diet was increased; similar changes were obtained in the groups with the same starch level, and most of the pathways were restrained in higher CP groups when compared with lower CP groups.

## 5. Conclusion

This study found that heart girth gain (d 180) and BUN (d 60) was significantly affected by protein × starch (DPSRs). The level of ALB was significantly affected independently by protein and starch, respectively, on d 60 during the whole experimental period, the gastrointestinal microbiota were different among the dietary treatments. Compared with pre-weaning period, the rumen microbial abundance increased in post-weaning period, whereas the cecum microbial abundance was decreased. In a word, these results indicated that the BUN, ALB, and gastrointestinal microbiota responded distinctly to differing DPSRs or period, and a reasonable DPSRs for calves in different stages of development is important.

## Data availability statement

The datasets presented in this study can be found in online repositories. The names of the repository/repositories and accession number(s) can be found at: https://www.ncbi.nlm.nih.gov/, SRP348482.

## Ethics statement

The animals were managed according to the guidelines for the care and use of experimental animals of Jilin Agricultural University Care and Use Committee and commercial dairy farms (JLAU-ACUC2019-018, Changchun, China).

## Author contributions

YZ, ZS, TW, GQ, and XZ: conceptualization. YZ and XC: methodology. XC, ZH, JD, JX, and JR: formal analysis. XC, YZ, JX, WZ, NA, and ZS: data curation. GQ, YZ, TW, and ZS: supervision. XC and ZH: writing—original draft. XC, YZ, ZS, and TW: writing—review and editing. All authors contributed to the article and approved the submitted version.

## Funding

This study was supported by the National Natural Science Foundation of China (grant number 32102574) and Scientific and Technological Developing Scheme of Jilin Province (grant numbers 20210202037NC and 20220202049NC).

## Conflict of interest

YZ and ZS were employed by Changchun Borui Science and Technology Co., Ltd.

The remaining authors declare that the research was conducted in the absence of any commercial or financial relationships that could be construed as a potential conflict of interest.

## Publisher’s note

All claims expressed in this article are solely those of the authors and do not necessarily represent those of their affiliated organizations, or those of the publisher, the editors and the reviewers. Any product that may be evaluated in this article, or claim that may be made by its manufacturer, is not guaranteed or endorsed by the publisher.

## Footnotes

^1^https://metacyc.org/
